# Chemsex, Sexual Health and Community-Based Mental Health Approaches: A Narrative Review

**DOI:** 10.1192/j.eurpsy.2026.10779

**Published:** 2026-07-31

**Authors:** Â. Ferreira, M. Cunha, G. Lacerca, J. Miranda, J. Marta, C. Batista, D. Durães

**Affiliations:** Arrábida’s Local Health Unit, Setúbal, Portugal

## Abstract

**Introduction:**

Chemsex — the intentional use of psychoactive substances to facilitate or enhance sexual activity, particularly among men who have sex with men (MSM) — is an emerging public health and mental health concern across Europe. It is strongly associated with risky sexual practices, increased prevalence of sexually transmitted infections (STIs), HIV, and significant psychiatric comorbidities, including anxiety, depression and substance use disorders. Traditional individual-focused interventions have had limited reach, highlighting the need for broader community-based strategies.

**Objectives:**

To examine the intersection between chemsex, mental health and community responses, exploring how integrative, community-driven approaches can mitigate harms and enhance resilience in affected populations.

**Methods:**

Narrative synthesis of European studies (2015–2024) on chemsex prevalence, associated harms, and mental health outcomes. Review of community-based interventions, including peer-led initiatives, sexual health services, and integrated addiction/mental health programmes.

**Results:**

Chemsex prevalence among MSM varies between 3% and 29% across European settings. Users present higher rates of STIs and psychiatric morbidity compared to non-users. Harm reduction and peer-support interventions show promising outcomes in reducing risky behaviours and improving mental health engagement. Integration of sexual health, addiction and psychiatric services within community-based frameworks enhances accessibility, reduces stigma, and fosters a sense of belonging and empowerment.

**Conclusions:**

Chemsex must be addressed as both a clinical and societal issue. Community-based, multidisciplinary interventions — combining sexual health, addiction treatment and psychosocial support — are essential to reduce harm and promote holistic recovery. Embedding chemsex responses within broader community psychiatry aligns with the congress theme of a “whole society approach,” fostering inclusion, prevention, and shared responsibility.

**Disclosure of Interest:**

None Declared

